# Sustainable Photovoltaic-Powered Series Electrochemical Reactors Using Nonactive Electrocatalytic Materials for Electro-Refinery in Organics and Energy Production

**DOI:** 10.1021/acsomega.5c01329

**Published:** 2025-07-14

**Authors:** Rainy Alves de Sousa, Livia N. Cavalcanti, Amanda D. Gondim, Jussara Câmara Cardozo, Marco A. Quiroz Alfaro, Carlos A. Martínez-Huitle, Elisama Vieira dos Santos

**Affiliations:** † Renewable Energies and Environmental Sustainability Research Group Institute of Chemistry, Federal University of Rio Grande do Norte, Campus Universitário, Av. Salgado Filho 3000, Lagoa Nova, Natal, Rio Grande do Norte CEP 59078-970, Brazil; ‡ Human Resources Program of the National Agency for Petroleum, Natural Gas and Biofuels − PRH-26-ANP, Graduate Program in Chemical Engineering - PPGEQ, Lagoa Nova, Natal/RN 59078-970, Brazil; § National Institute for Alternative Technologies of Detection, Toxicological Evaluation and Removal of Micropollutants and Radioactives (INCT−DATREM), Institute of Chemistry, UNESP, P.O. Box 355, Araraquara, SP 14800 900 Brazil

## Abstract

The applicability of an electrochemical device with a series design and configuration, powered by photovoltaic panels, was tested as an integrated-hybrid approach for energy-efficient electrochemical green H_2_ production coupled with dye effluent upgrading. This electrochemical strategy investigated the electroconversion efficiencies of a model organic compound into carboxylic acids by applying 15, 30, and 45 mA cm^–2^, with simultaneous production of green H_2_. Fluorine-doped lead oxide (PbO_2_–F) and boron-doped diamond (BDD) electrodes were used as anodes in a three-compartment flow reactor, in series mode with different anodic order: setting 1 (PbO_2_–F + BDD) and setting 2 (BDD + PbO_2_–F). PbO_2_–F was successfully prepared using the electrodeposition method, and it was characterized. The results clearly showed that configuration 1 (PbO_2_–F + BDD) consumed 10.47 kWh m^–3^ after 120 min of electrolysis by applying 45 mA cm^–2^, achieving 42.5% organic removal efficiency and producing simultaneously 0.7 L of green H_2_. Meanwhile, the configuration 2 (BDD + PbO_2_–F) consumed 16.18 kWh m^–3^ under the same operating conditions, achieving 47.5% organic load removal and producing 0.75 L of green H_2_. Discoloration of the effluent was efficiently achieved in all cases. Carboxylic acid production was monitored over time at different conditions tested. Notably, a selective production of malonic acid (28% conversion efficiency) was attained using setting 1, and consequently, upgrading the electro-refinery concept using wastewater residues.

## Graphical abstract

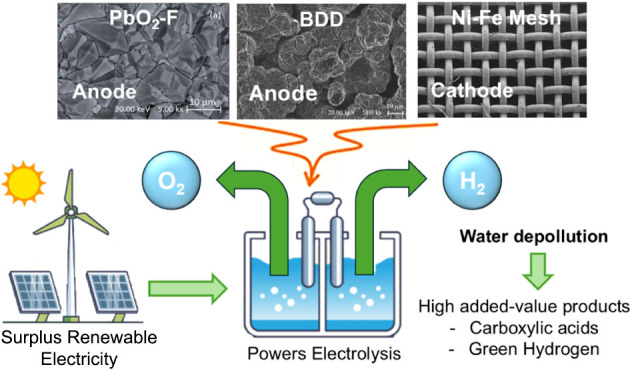

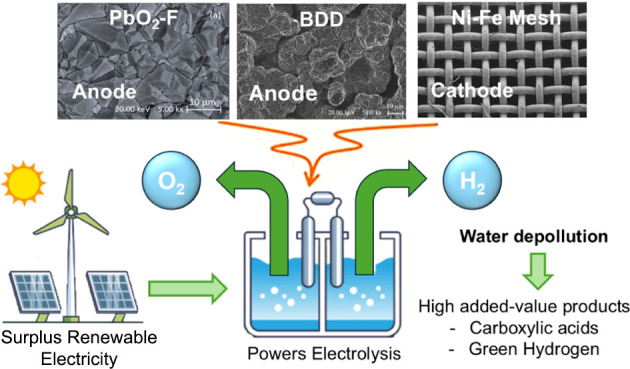

## 1 Introduction

The treatment of wastewaters containing dissolved recalcitrant pollutants by electrochemically assisted techniques, aiming at the mineralization of such pollutants, has been a hot topic receiving great attention for several years.^1,2^ Nevertheless, society is now aware that treating waste, as was the environmental paradigm several decades ago, is no longer sufficient. It is necessary to go further with treatment objectives, seeking more sustainable solutions that help mitigate major environmental issues emerging on Earth, including global warming and the exhaustion of natural resources.^3,4^ Thus, modern society is on the way to changing the current waste treatment concept by the paradigm of a circular economy, in which waste is used to recover raw matter, avoiding their exhaustion from Earth and contributing positively to the decrease in the carbon and water footprint of both production and environmental technologies.^5^ A promising starting point in agreement with the circular economy principles is a strategy that remains underexplored with electrochemical technologies for water treatment but is the premise of the electro-refinery concept, in which electrochemical waste valorization offers a sustainable alternative for obtaining valuable fine chemicals, e.g., carboxylic acids, and energy sources, e.g., hydrogen.^6,7^

Electrochemical oxidation (EO) is experiencing a renaissance as an electrocatalytic approach for promoting waste valorization, primarily due to its technological simplicity, sustainability, and control conditions.^8^ The nature of the anode, where EO occurs, strongly influences process efficiency as well as mineralization or conversion pathways.^9^ Currently, the anodic materials most commonly used in EO are boron-doped diamond anodes,^10,11^ noble metal anodes,^8,12^ and metal oxide anodes (SnO_2_,^13^ PbO_2_ ^14^). The PbO_2_ electrode is considered an excellent metal oxide electrode due to its high chemical stability in corrosive media, relatively high overpotential in oxygen evolution reactions, and low cost compared with noble metal electrodes. Also, certain elements can be added to the PbO_2_ layer,^15^ such as Co,^16^ Bi,^17,18^ Cu,^19^ Ce,^20^ Ni,^21^ and F,^22–24^ increasing its efficiency in oxidizing organic compounds. On “nonactive” electrodes, like PbO_2_, SnO_2_ and BDD, physically adsorbed active oxygen species (mainly hydroxyl radicals (^•^OH)) participate directly in organic oxidation, favoring complete mineralization (CO_2_ + H_2_O) or the conversion of pollutants.^25–27^ However, most of these anodes present versatile behavior in practice, and both processes, mineralization and electrochemical conversion, can occur in water matrices.^28^

The efficiency of EO also depends on the electrochemical reactor, which is also an essential factor in the real applicability of this technology. Special attention should be paid to the reactor design and configuration to achieve either high pollutant removal or highly selective conversion.^29,30^ There is a wide range of reactor engineering designs employed in electrochemistry, which can be divided or nondivided cells, batch or flow,^31–33^ and series flow electrochemical reactors^34,35^ or divided membrane-type reactors.^36–41^ In this context, a good design of electrochemical reactors and a good selection of reactor type allow for the optimization of the mass transport coefficient, the volume of effluent to be treated, and the (electro)­chemical reactions to be promoted. Within this framework, the effectiveness of electrochemical technology is influenced by the system configuration and can be altered by factors such as turbulence promoters, geometrical configuration, hydrodynamics, current density, electrolyte type, membranes, electrode selection, and so on, as reported in the existing literature.^30,42^

In the assessment of novel materials, electrochemical reactors, and wastewater treatment processes, dyes are frequently employed as standard pollutants due to their renowned reactivity toward oxidants produced during electrolysis. Furthermore, due to their straightforward and representative chemical composition, dyes are among the most important model pollutants in water treatment research. A variety of synthetic dyes, including Rhodamine B, Acid Orange II, Reactive Black 5, Remazol Red, Alizarin Blue Black B, Indigo Carmine, Malachite Green, Methyl Orange, Acid Red 211, and Procion Blue, as well as real textile effluents, have been studied under different levels of current densities (3.1 – 100 mA cm^–2^), reactor configuration (single or couple reactors), temperatures (25 – 60 °C), electrolytes and flow rates (60 – 720 L h^–1^).^13,43–45^ These treatments resulted in the effective removal of color and organic matter (in terms of chemical oxygen demand (COD) and total organic carbon (TOC)) by more than 90%, while maintaining reasonable operating costs.^46,47^

In this context, the aim of this work was to investigate the performance of a divided membrane-type flow reactor in series mode, powered by an energy source connected to photovoltaic (PV) panels. The divided membrane-type flow reactor used BDD and PbO_2_–F as anode electrodes, and a Ni–Fe-based material as the cathode for the treatment of a 1 L solution containing 20 mg L^–1^ methyl orange (MO) dye, chosen as a model organic compound due to its dark orange color, high solubility in water, and widespread use in textile industries.^48^ The process was investigated by analyzing the effect of different experimental conditions, such as applied current density (*j* = 15, 30, and 45 mA cm^–2^) and electrode arrangements (BDD and PbO_2_–F) on the organic matter removal, selective conversion into byproducts, and the production of green H_2_. To evaluate the level of degradation of the dye, spectrophotometric, electroanalytical (differential pulse voltammetry), and COD analyses were performed to follow the color, concentration, and organic load of the pollutant, respectively, while carboxylic acid production was monitored by ion chromatography (IC) to identify the final byproducts formed.

## 2 Materials and Methods

### 2.1 Reagents and Materials

MO (puriss. p.a. 99.5%) and H_2_SO_4_ (puriss. p.a. 95–97%) were sourced from Neon companies. Sigma-Aldrich’s Nafion membrane was used in all experiments. Aqueous solutions of potassium ferri/ferrocyanide (K_3_Fe­(CN)_6_/K_4_Fe­(CN)_6_) ranging from 20 to 80 mmol L^–1^ in 0.5 mol L^–1^ NaOH were also prepared to electrochemically characterize the electrochemical reactor. The model organic compound solution was prepared by adding 20 mg L^–1^ MO to a 0.05 mol L^–1^ Na_2_SO_4_ solution, while the cathodic compartment contained only the 0.05 mol L^–1^ Na_2_SO_4_ solution. Water purified using a Milli-Q system, with resistivity ≥ 18 MΩ cm at 25 °C, was obtained.

### 2.2 Preparation of F-Doped PbO_2_ Films on Ti Substrate

To prepare PbO_2_–F films, a titanium (Ti) plate was used as a support, which was pretreated to guarantee fixation of the metal oxide film by applying a methodology described elsewhere.^49^ Subsequently, electrodeposition was carried out in a solution containing 0.25 mol L^–1^ Pb­(NO_3_)_2_, 0.1 mol L^–1^ HNO_3_, and 0.01 mol L^–1^ NaF, applying a current density of 5 mA cm^–2^ for 2 h, in a batch system, using a magnetic bar for stirring the solution. The Ti plate was placed as the anode and a stainless-steel (SS) plate as the cathode.^22^ The geometric area of the Ti/PbO_2_–F electrode was 8.4 cm^2^. For more information about the characterization of the electrodes produced, as well as the analytical techniques used, see the Supporting Information.

### 2.3 Flow Cell Configuration

A divided membrane-type flow reactor, in series mode, was used for degrading 1 L of 20 mg L^–1^ MO dye solution in 0.05 mol L^–1^ Na_2_SO_4_ and simultaneous green H_2_ production (see Figure 1). BDD (8.4 cm^2^) and synthesized PbO_2_–F (8.4 cm^2^) were used as anodes and arranged in two anodic compartments, while a Ni–Fe-based SS as the cathode was placed in the cathodic reservoir. The configuration of the flow electrochemical reactor consists of three compartments, two anodic and one cathodic, separated by two Nafion 350 membranes, as described in Figure 1b. Electrolysis of 1 L of synthetic solution containing 20 mg L^–1^ MO dye in 0.05 M Na_2_SO_4_ as the supporting electrolyte was recirculated through the two anode compartments (Figure 1a), while 65 mL of 0.25 mol L^–1^ H_2_SO_4_ were inserted into the cathodic compartment and remained stationary without flow. H_2_ generated in the cathodic compartment was collected using a distilled water protocol.^13^ The reactor was assembled with acrylic parts featuring predrilled holes for the inlet and outlet of the solution and for the electrical connections (Figure 1b). Considering the reactor arrangement, the impact of the arrangement of the two anodes^29,50^ in the cell was examined:

**Figure 1:**
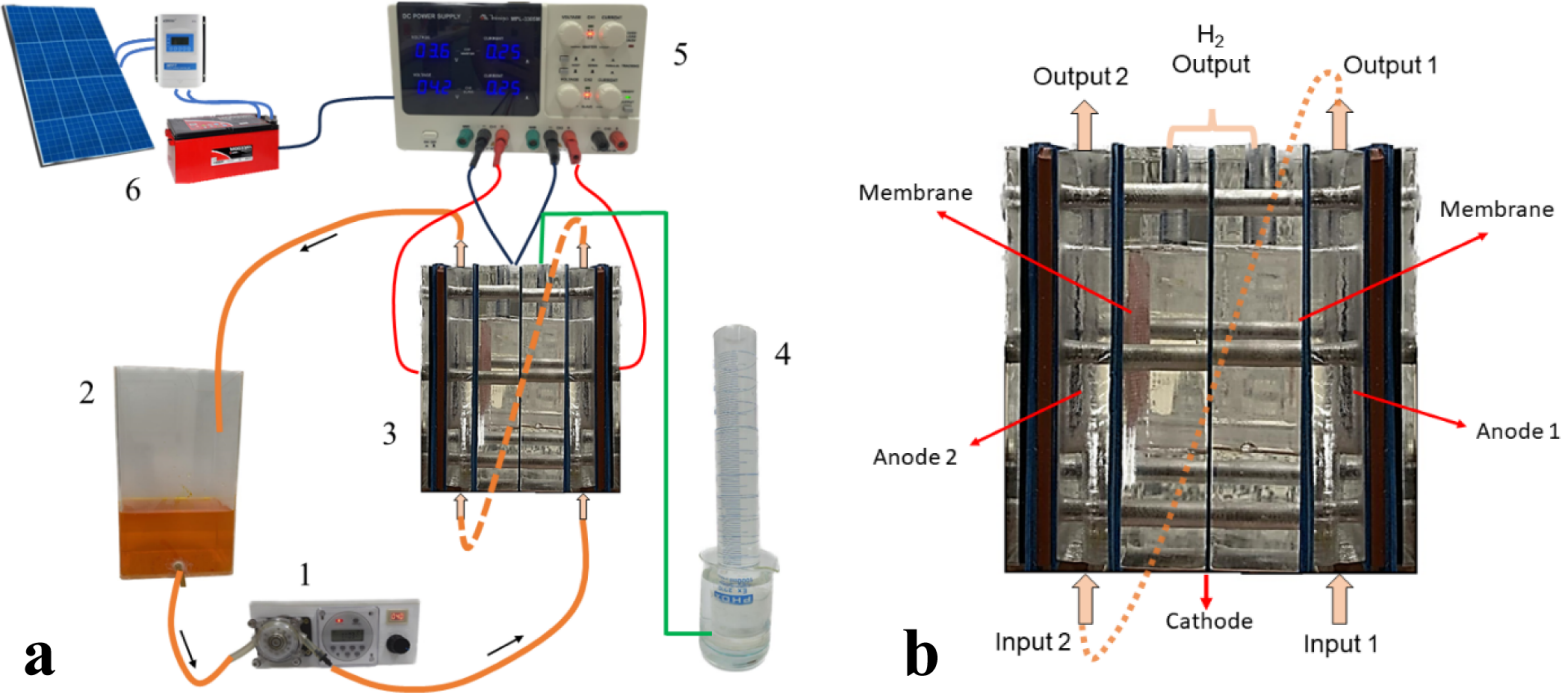
(a) Schematic diagram of the complete electrochemical system: (1) peristaltic pump, (2) reservoir of effluent (volume: 1 L), (3) divided membrane-type flow reactor, (4) H_2_ collector, (5) power supply, and (6) solar PV – battery power system. (b) Divided membrane-type flow reactor details.

In configuration 1, the solution was recirculated through the first anodic compartment, in which the synthetic effluent was electrolyzed by the PbO_2_–F anode, and subsequently in the second anodic reservoir (see, Figure 1) where the electrolysis was followed by the BDD anode.

In configuration 2, the order of the recirculation of the effluent was modified. First, the effluent was electrochemically treated at the anodic compartment with a BDD electrode, and afterward, the effluent was recirculated through the second anodic reservoir containing PbO_2_–F electrode (Figure 1).

Thus, for both configurations, the experiments were carried out at different *j* (15, 30, and 45 mA cm^–2^) (see Table 1).

**Table 1:** Comparison of Selected Anodes (BDD and PbO_2_) and Electrode Arrangement in the Electrochemical Cell with Respect to the Production of Carboxylic Acids

					[Table-fn t1fn1-fo]139.4		Conversion selectivity/%[Table-fn t1fn3-fo]					
Entry	Setting	E_cell_/V	*k*/min^–1^	R_COD_/%	Color DFZ_464_ [Table-fn t1fn2-fo]	Rate H_2_/mmol min^–1^	Acetic	Formic	Malonic	Tartaric	Salicylic	Total
	setting 1 (PbO_2_-F + BDD)											
1	15 mA cm^–2^	13.86	0.059	30	1.10	0.086	1.09	0.47	13.21	1.71	0.21	16.69
2	30 mA cm^–2^	17.67	0.103	37.5	0.90	0.16	3.64	3.67	24.98	3.72	0.34	36.35
3	45 mA cm^–2^	22.70	0.184	42.5	0.50	0.23	0.58	1.10	28.00	1.97	0.42	32.07
	setting 2 (BDD + PbO_2_-F)											
4	15 mA cm^–2^	13.55	0.042	35	1.20	0.089	0.22	0.59	8.99	0.93	0.01	10.74
5	30 mA cm^–2^	21.63	0.090	37.5	0.90	0.15	0.25	0.71	24.24	2.29	0.35	27.84
6	45 mA cm^–2^	22.55	0.240	47.5	0.10	0.24	0.36	1.54	25.94	1.83	0.25	29.92

a Color DFZ_464_ value before treatment,

b Color DFZ_464_ values after treatment,

c Conversion selectivity (%) = yields, based on %C referred to the initial TOC (1.51 mg L^–1^) of the MO,

To monitor the performance of the electrochemical tests, analytical determinations were carried out, such as differential pulse voltammetry (DPV, see Section 3 in the SI and Figure S1), COD, ion chromatography, inductively coupled plasma atomic emission spectroscopy (ICP-AES), and UV–vis spectrophotometry, as in Sections 2 and 3 in the SI.

## 3 Results and Discussion

### 3.1 Surface Characterization and Voltammetric Behavior of PbO_2_-F Electrode

SEM was used to characterize the morphology and surface structure of the PbO_2_–F/electrode. As shown in Figure 2a, the PbO_2_–F coating prepared by electrodeposition in the presence of F^–^ anions allows the growth of the PbO_2_ deposit with a more regular morphology and the formation of tetragonal crystallites, which are typical of PbO_2_ deposited from fluoride-containing solutions. This result agrees with other reports in the literature.^51^ The corresponding EDS spectrum of the PbO_2_–F electrode is shown in Figure 2b, which clearly shows the lead peaks and confirms the presence of fluorine. However, the atomic percentage of fluorine on the surface was about 2.9%, because the concentration of F^–^ anions in the electrolyte was lower. Meanwhile, XRD patterns of the PbO_2_–F electrode are shown in Figure 2c, and all diffraction peaks are strong and sharp, indicating that the PbO_2_–F anode obtained was well-crystallized.^52^ It can also be seen in the XRD analysis that the electrode predominantly has the peaks that characterize the desired β-PbO_2_–F phase at angles 31.6°, 35.9°, 48.8°, 62.3°, 66.5°, 77.4°, 83.7°, and 85.7° assigned to the (101), (200), (211), (301), (202), (400), (312), and (411) planes.^53,54^ However, a small fraction of peaks also corresponded to phase α, which were observed at 2θ = 43.8° and 64.2°.^52^ α and β forms determine the electrocatalytic activities of PbO_2_ surfaces; therefore, these forms have been widely studied to understand their effect on the degradation of different organic compounds, electrochemical generation of ozone, wastewater treatment, electrosynthesis, and other processes.^54,55^ β-PbO_2_ is considered more active and conductive than α-PbO_2_ due to its higher oxygen overvoltage.^56–58^ Therefore, β-PbO_2_ surfaces are generally used for several electrochemical applications.

**Figure 2:**
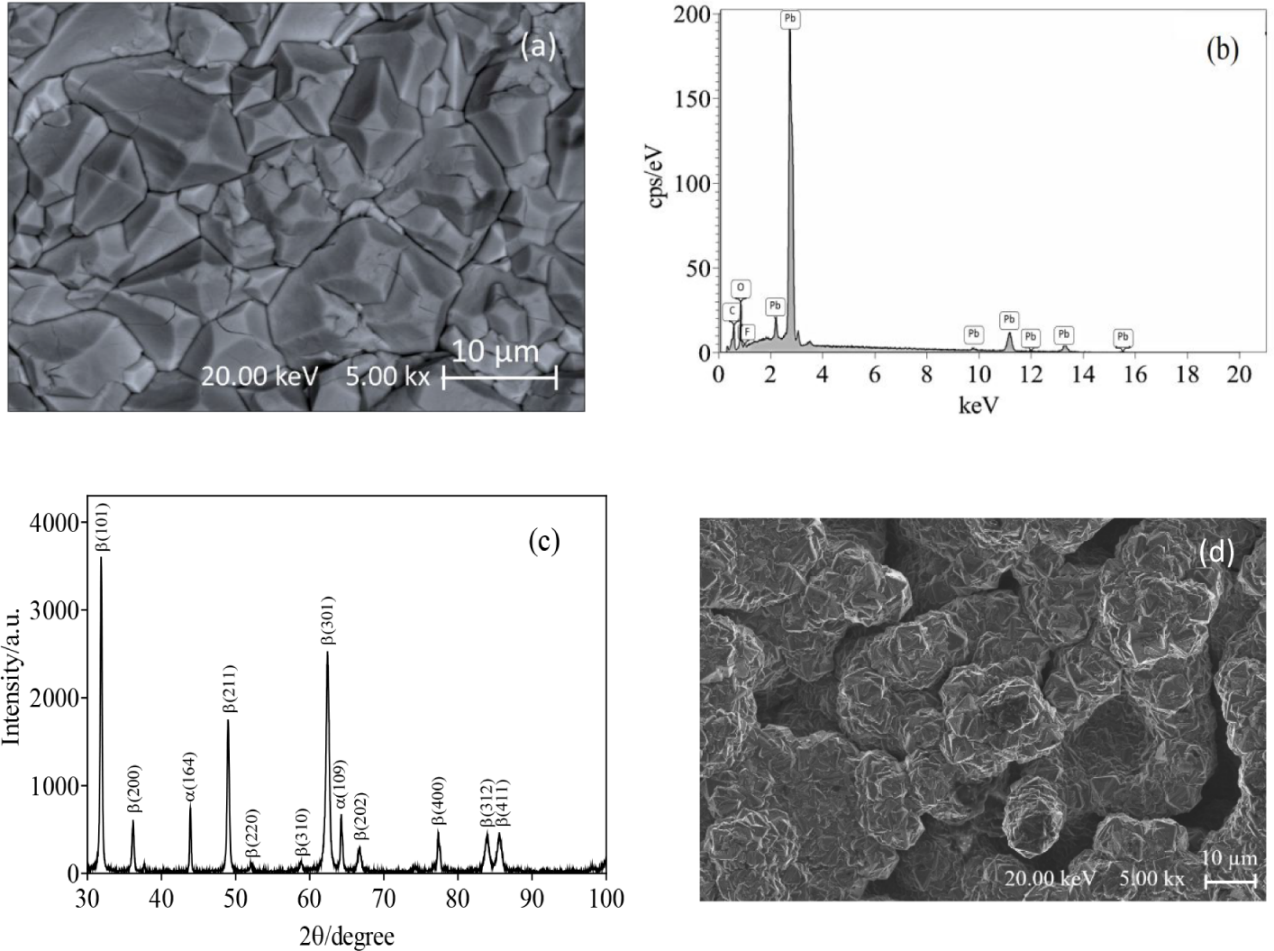
(a) Scanning electron micrograph (SEM) and (b) EDS spectra of PbO_2_–F/Ti electrode; (c) XRD patterns of PbO_2_–F/Ti electrode; and (d) SEM of BDD electrode.

In the case of the BDD film, the SEM image is shown in Figure 2d. It was noted that the film represents a dominant morphological aspect formed by microcrystalline grains randomly oriented and associated with the polycrystalline diamond film, which agrees with the results reported in the literature.^59^

The oxygen evolution activity of the PbO_2_–F and BDD electrodes was evaluated by using linear polarization voltammetry measurements in a 0.05 mol L^–1^ Na_2_SO_4_ solution at a scan rate of 50 mV s^–1^. The voltammetric profiles showed that the oxygen evolution potential (OEP) for PbO_2_–F is about +1.7 V and for BDD is approximately +1.9 V relative to Ag/AgCl (3 mol L^–1^), see Figure 3. These values align with the results published by other authors, where the values of the OEP are similar.^52,54,60^ Normally, the inhibition of the OER is desired for the application of the electrode in the field of environmental electrochemistry^61,62^ because the electrogeneration of physically absorbed ^•^OH is promoted at higher concentrations, which favors the degradation of organic compounds. As can be seen in Figure 3, the OER is inhibited at higher current densities when the BDD electrode is used.^63,64^ Tafel plots (inset in Figure 3) for PbO_2_–F and BDD electrodes, at lower and higher overpotential regions, evidenced that the formation of hydroxyl radicals is attained at both materials, but it is not the only main mechanism at the BDD electrode in the sulfate supporting electrolyte. Analyzing the Tafel slopes, sulfate-based oxidant production, such as persulfate and sulfate ion radicals (data reported in the inset of Figure 3), has significant importance that over hydroxyl radicals, mainly at the BDD anode. This behavior was already studied and confirmed by our group, where at higher sulfate concentrations, sulfate ion radicals and persulfate are formed by indirect mechanism involving the participation of hydroxyl radicals, as discussed below.^65–70^

**Figure 3:**
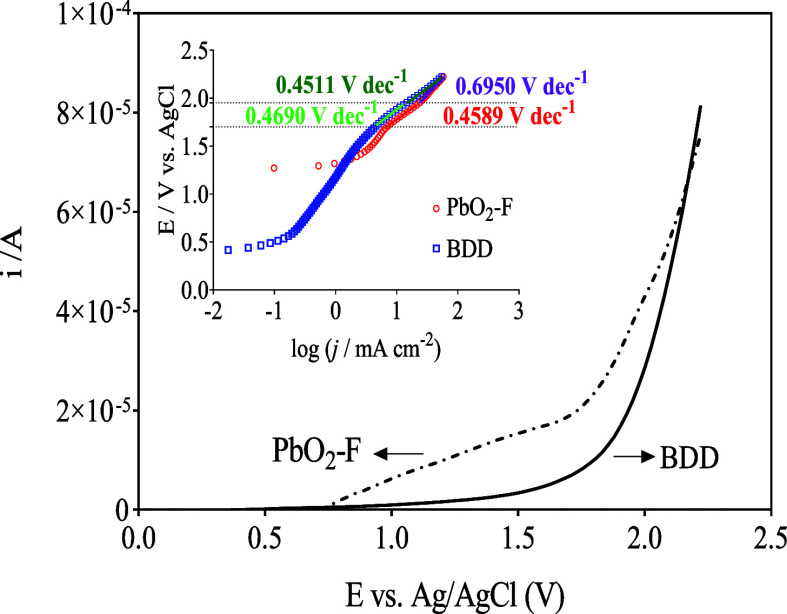
Linear polarization curves (second scan reported) recorded in 0.05 mol L^–1^ Na_2_SO_4_ at 25 °C and scan rate of 50 mV s^–1^, for the PbO_2_–F/Ti and BDD electrode, respectively. Inset: Tafel plots at lower and higher overpotential regions from the potential of hydroxyl radical, persulfate, and oxygen formation.

### 3.2 Electrochemical Oxidation of MO: Effect of Arrangement Electrode Combinations and Current Density

#### 3.2.1 Mass Transport

The assembly of electrochemical flow cells in series for removing dyes has already been investigated by our group.^29,71^ Therefore, the arrangement combinations tested here (nature of electrode – active or nonactive – cell-electrode positions, current densities, organic removal load, and so on) have been chosen based on our previous investigations.

Initially, the divided membrane-type flow reactor (see Figure 1), in series mode, was characterized in order to estimate the mass-transfer coefficient (*k*_m_) by the K_3_Fe­(CN)_6_/K_4_Fe­(CN)_6_ protocol^72^ using eq S2 in Section 4 in the SI. Polarization curves (current vs potential (see Figure S2)) were plotted for each studied concentration (1–10 mmol L^–1^ in 0.5 mol L^–1^ NaOH, which were recirculated into the anodic compartments with a constant flow rate of about 167 mL min^–1^) to determine the diffusion-limited current, which was then used to construct the calibration curve (inset in Figure 2S). A linear relationship was observed between the studied concentrations and the corresponding limit current with a high correlation (*R* ^2^ = 0.9971). Using the angular coefficient of the line (limit current vs concentration), the experimental mass-transfer coefficient (*k*_m_) was calculated, yielding a value of 9.45 × 10^–5^ m s^–1^. This analysis is crucial for understanding the diffusive phenomena within the system and, based on these insights, minimizing physical resistances to achieve higher diffusion efficiency. Then, the result indicates that the reactor design facilitates the efficient movement of molecules/ions towards the electrode surface during electrolysis. In fact, the *k*_*m*_ value obtained here was slightly superior to that of the single cell, which is about 8.58 × 10^–5^ m s^–1^.^73^ For this reason, the use of this arrangement could favor an efficient elimination of organic compounds from water matrice.^75–76^ The value of *k*_*m*_ was subsequently used together with the initial value of the COD of the MO effluent, as required by eq S3, to estimate the limiting current (*I*_lim_), which was 0.06 A (*j* = 7.64 mA cm^–2^). This value is comparatively lower than the current values of 0.13, 0.25, and 0.38 A corresponding to the current densities of 15, 30, and 45 mA cm^–2^, respectively. This result suggests that oxidation under these conditions occurs by mass transport control. These assumptions agree with the studies recently published^77,78^ during the anodic oxidation of other wastewaters.

#### 3.2.2 Effluent Discoloration and Degradation of Organic Matter

The behavior of the MO concentration decay (1 L of 20 mg L^–1^ MO in 0.05 mol L^–1^ Na_2_SO_4_) and color removal efficiencies, as a function of electrolysis time, when both cell configurations were used by applying different *j*, is depicted in Figure 4. Analyzing the results obtained according to the experimental conditions reported in Table 1, MO was completely removed from the synthetic solution at all *j* values tested. However, the removal rate depended on the *j*, achieving a complete elimination of the dye, in both arrangements, at approximately 90, 60, and 30 min for 15, 30, and 45 mA cm^–2^, respectively.

**Figure 4:**
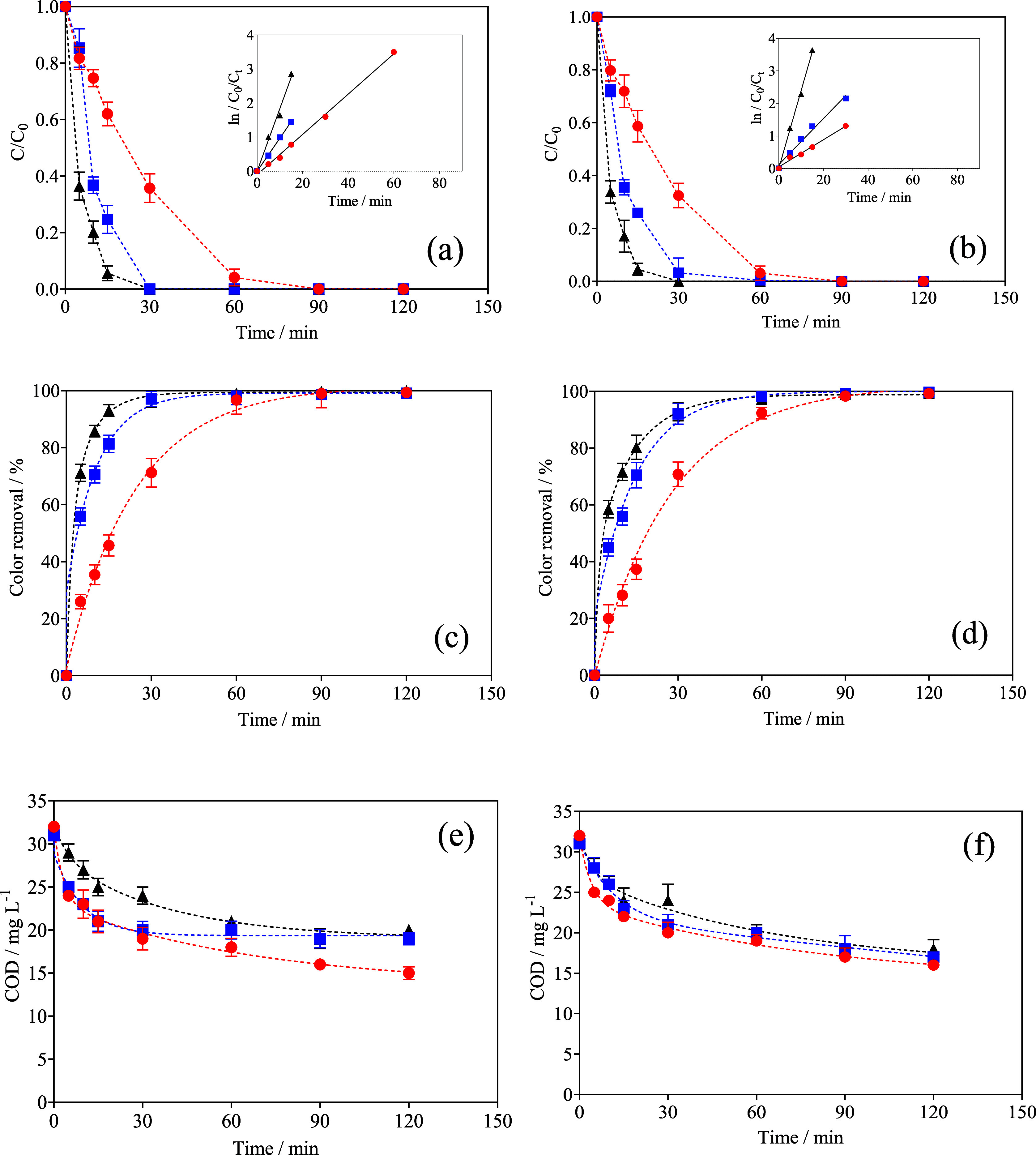
Electrochemical treatment of MO with a divided membrane-type flow reactor, in series mode ((a,c,e) setting 1 (PbO_2_–F + BDD) and (b,d,f) setting 2 (BDD + PbO_2_–F)) driven by a solar PV-battery system, applying different *j* ((●) 15, (■) 30 and (▲) 45 mA cm^–2^). (a,b) MO concentration decay over time using different cell assembling in series mode at different *j*. Inset: kinetic oxidation plots for the effect of *j*. (c,d) Effect of the *j* on the color removal efficiencies, as a function of electrolysis time. (e,f) COD decay as a function of time of electrolysis with different cell arrangements. Operating conditions: 0.05 mol L^–1^ Na_2_SO_4_ as electrolyte; 20 mg L^–1^ of MO as initial concentration, and 1 L of synthetic effluent that was recirculated by the divided membrane-type flow reactor at 25 °C to degrade MO and simultaneously produce green H_2_.

As can be observed in the inset of Figures 4a,b, a pseudo-first-order kinetic model was followed by the elimination of dye from the solution in all cases (Section 5 in the SI). Then, satisfactory linear tendencies were achieved at all *j*, exhibiting regression coefficients greater than 0.97, by fitting a pseudo-first-order reaction. These kinetic results allowed us to determine that setting 1 (PbO_2_–F + BDD) promoted a fast MO elimination from the solution, reaching rate constant (*k*) values of approximately 0.057 min^–1^ (*R* ^2^ = 0.985, 15 mA cm^–2^), 0.103 min^–1^ (*R* ^2^ = 0.987, 30 mA cm^–2^), and 0.184 min^–1^ (*R* ^2^ = 0.990, 45 mA cm^–2^). Meanwhile, setting 2 (BDD + PbO_2_–F) presented *k* values of about 0.042 min^–1^ (*R* ^2^ = 0.988, 15 mA cm^–2^), 0.090 min^–1^ (*R* ^2^ = 0.997, 30 mA cm^–2^), and 0.24 min^–1^ (*R* ^2^ = 0.996, 45 mA cm^–2^). Then, the behavior of the degradation rates related to the oxidative performances of each one of the settings investigated depends on the production of oxidants and the capacity of them to react with the organic compounds. In the case of setting 1 (PbO_2_–F + BDD), it achieves higher degradation rates by applying 15 and 30 mA cm^–2^ than those attained in setting 2. Conversely, setting 2 (BDD + PbO_2_–F) is superior to setting 1, in terms of degradation rate, when higher *j* is applied to the electrochemical system (45 mA cm^–2^). It is associated with the most efficient production of hydroxyl radicals and persulfate on the BDD surface by applying higher *j*, as already confirmed by the voltammetric results in Figure 3. It is important to consider that the production of ^•^OH has a key role in dye decomposition efficiency,^79^ as does the in situ electrosynthesis of secondary oxidants. At nonactive anodes (e.g., BDD and PbO_2_), water discharge is expected to be the predominant anodic process, resulting in the formation of free ^•^OH that can react with organic compounds present in the effluent (eqs 1 and 2),^79^ in this case, MO.

BDD⁡+⁡H2O→BDD(OH•)+H++e− 1

PbO2+H2O→PbO2(OH•)+H++e− 2

Therefore, MO concentration quickly decays to zero, in all cases, when different *j* values are applied (see Figure 4a,b).

On the other hand, the type of electrolyte has also made a significant contribution to the electrochemical incineration or conversion of organic matter due to the anodic electrosynthesis of oxidants. Based on the existing literature, the electrogeneration of sulfate radicals (eq 3) and persulfate (S_2_O_8_^2–^) (eqs 4 and 5) is feasible on PbO_2_ and BDD electrodes when a sulfate-based electrolyte is used,^67,80,81^ as in this study. These sulfate-based oxidant species enhance the oxidation of organic matter by radical and nonradical mechanisms.^67^

$${{\mathrm{SO}}_{4}}^{2-}\to {{\mathrm{SO}}_{4}}^{•-}+e^{-}$$ 3

4 $$2{{\mathrm{SO}}_{4}}^{2-}\to S_{2}{O_{8}}^{2-}+2e^{-}$$

5 $${{\mathrm{SO}}_{4}}^{•-}+{{\mathrm{SO}}_{4}}^{•-}\to S_{2}{O_{8}}^{2-}$$

These oxidants promote the fragmentation of the chromophore group of dye, as first step, and subsequently, the production of byproducts that are also degraded close to the anodic surface or in the bulk. Since the ability to generate oxidants at nonactive anodes depends on the *j*, the results reported in Figure 4 related to the MO decay (Figure 4a,b) can be attributed to the increase in the concentration of •OH and sulfate-based oxidant species.

In the case of discoloration (Figure 4c,d), the decrease of the absorption band of the MO solution indicated a rapid degradation of the azo group, consequently causing the elimination of color in the synthetic effluent. It was clear that complete discoloration is also independent of the *j* and configuration setting. Conversely, the elimination of color is achieved at different times depending on the configuration of the cell ((PbO_2_–F + BDD) and (BDD + PbO_2_–F)) and *j* applied. Then, these trends follow similar behavior achieved when the MO concentration was monitored (Figure 4a,b). Discoloration is quickly attained at lower *j* values in setting 1, while setting 2 (BDD + PbO_2_–F) is superior when a higher *j* is applied to the electrochemical system (45 mA cm^–2^), which is associated with direct and indirect EO processes. Thus, PbO_2_–F electrode surface’s interaction with unpaired electrons from the nitrogen atoms in the azo group (−N=*N*−) promotes the direct oxidation process. Conversely, at high *j*, indirect EO is favored, and the level of degradation significantly increases. The discoloration process of 1 L of 20 mg L^–1^ MO allowed us to estimate the DFZ (DurchsichtFarbZahl, visual color number in German, as indicated in eq S1 in the SI) parameters for each operating condition, and consequently, it was possible to establish the wastewater quality for plausible reuse. The initial MO effluent showed a color with a DFZ_464 nm_ of 139.4 m^–1^; however, for reuse requirements in textile processing or disposal of this effluent, the decolorization is needed, i.e., nonvisible color, and then, the initial value should be substantially diminished. In this context, when DFZ values were assessed at the end of the electrolysis for both series cell configurations (setting 1 (PbO_2_–F + BDD) and setting 2 (BDD + PbO_2_–F)), 0.5 m^–1^ and 0.1 m^–1^ estimations were reached, respectively. In that case, setting 2 had the best efficiency result in decolorizing the effluent.

Figure 4e,f shows that the electrochemical treatment of 1 L of 20 mg L^–1^ of MO in Na_2_SO_4_ 0.05 mol L^–1^ was achieved. In general, similar behaviors were observed during the organic matter removal, in terms of COD, by applying different *j* using both settings. The results indicate that COD was partially removed after 120 min of electrolysis at all *j* values, reaching a maximum of about 42% and 47% at 45 mA cm^–2^, in configurations 1 and 2, respectively. These behaviors can be associated with operating regimes, which are a result of the mass transport effects, deactivation of nonactive electrode surfaces,^76^ competition with oxygen evolution,^79^ and the accumulation of intermediates that are difficult to degrade.

These operating regimes, governed either by charge or by mass transport, depend on the *j* or *i* applied. In the former case, electrolysis is regulated by *i*, achieving 100% current efficiency, and produces a linearly decreasing pollutant concentration over time (*i*_appl_ < *i*_lim_). Meanwhile, in the latter case, electrolysis is regulated by mass transport, where secondary reactions (including the oxygen evolution reaction) can be considered, leading to a reduction in current efficiency and resulting in an exponential trend in organic matter removal under these circumstances.

The limiting current value of about 0.06 A (7.64 mA cm^–2^) was estimated by considering the *k*_m_ of the reactor cell (*k*_m_ = 9.45 × 10^–5^ m s^–1^) together with the initial COD value for the synthetic MO effluent. This value is lower than the current values used in this work (0.13, 0.25, and 0.38 A for 15, 30, and 45 mA cm^–2^, respectively), which indicates that the degradation changes attained in the behavior of COD obey a mass transfer control. In fact, COD removals follow exponential behavior under all conditions, as can be observed in Figure 4e,f, until 120 min of electrolysis. These effects are a consequence of secondary reactions, such as the OER and oxidant electrosynthesis, as well as the byproducts formed, which could be more recalcitrant than the MO.^9^ Large amounts of •OH can result in the formation of O_2_, and this undesired reaction, in the wastewater treatment, avoids to achieve high organic matter removals^82^ (see Figure 4e,f). ^•^OH has a short lifetime with a mass transfer coefficient of approximately 10^–5^ m s^–1^, which makes it active only at the Nernst layer close to the anodic surface.^83^ Therefore, when the oxygen evolution reaction is favored (a high formation of oxygen bubbles at the anodic surface), the transport of the organic compound toward the electrode surface can be influenced,^79^ and consequently, the effectiveness of the process is affected.

### 3.3 Production of Persulfate

According to the voltammetric results and the existing literature, the electrogeneration of persulfate is feasible on PbO_2_ and BDD electrodes when a sulfate-based electrolyte is used,^80,81,84^ and these oxidants play a key role in the degradation of MO in solution. Therefore, the production of sulfate-based oxidants was monitored as a function of the system configuration and time when 0.05 mol L^–1^ Na_2_SO_4_ was used as the electrolyte by applying 15, 30, and 45 mA cm^–2^ (Figure 5). As can be seen in Figure 5, it was noted that the final concentration of S_2_O_8_^2–^ was about 0.23, 0.73, and 0.94 mg L^–1^ when setting 1 was used at 15, 30, and 45 mA cm^–2^, respectively. Meanwhile, concentrations of about 0.15, 0.56, and 1.30 mg L^–1^ persulfate were electrochemically produced at 15, 30, and 45 mA cm^–2^, respectively, after 120 min of electrolysis when setting 2 was used. It is important to indicate that the generation of S_2_O_8_^2–^ is formed by an indirect oxidation process where ^•^OH reacts with SO_4_^2–^ at the Nernst layer of nonactive electrodes, as already discussed above. Nevertheless, the BDD’s ability to produce sulfate-based oxidants is accentuated at higher *j* ^85^ (see also Figure 3 related to the polarization curves obtained for both anodic materials); for this reason, a more efficient production of these oxidants can be attributed to the use of arrangement 2 (see Figure 5) where the BDD anode is in the first anodic compartment. The trends described above clearly confirm the COD and color removal efficiencies obtained when each one of the cell’s arrangements is used.

**Figure 5:**
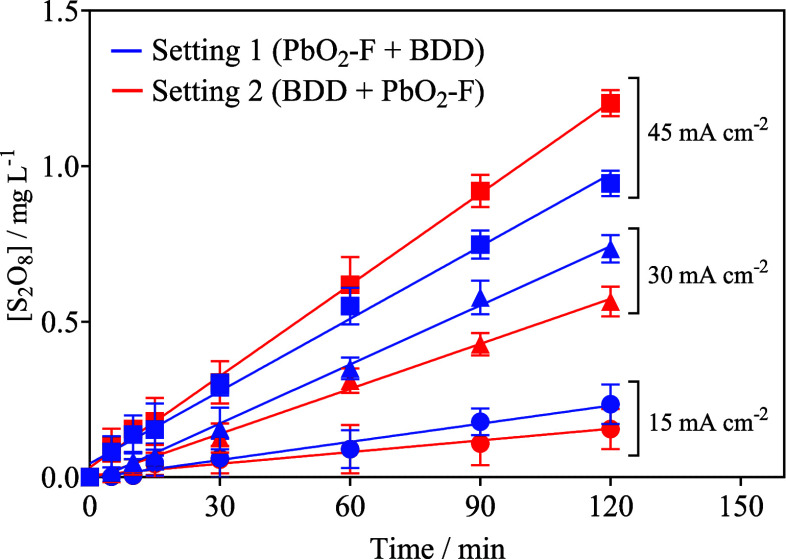
Electrochemical production of S_2_O_8_^2–^, as a function of reactor configuration and time, applying 15, 30, and 45 mA cm^–2^ in the presence of Na_2_SO_4_ 0.05 mol L^–1^.

### 3.4 Evolution of the Main Carboxylic Acids

To confirm the electrochemical degradation of MO into other compounds, the identification and quantification of final intermediates were performed. Nevertheless, the hybrid approach also emerges as a promising solution for the production and recovery of high value-added products in both electrochemical compartments (production of green H_2_ and organic compounds in the cathodic and anodic compartments, respectively) from the treatment of dyes.^41^ As shown in Figure 6, some carboxylic acids are produced by the fragmentation of the MO dye. A comparison of the trend in the concentration of carboxylic acids obtained in both cell arrangements showed a predominant generation of tartaric, malonic, and formic acids, and, to a lesser extent, acetic and salicylic acids. Thus, the configuration cell, *j*, and electrolysis time influence the production of these organic acids (see Table 1 and Figure 6). For example, an evaluation of the results indicates that formic, malonic, tartaric, and acetic acids reached 0.85, 9.78, 1.09, and 0.212 mg L^–1^, respectively, after 120 min of electrolysis when 15 mA cm^–2^ (Figure 6a) was applied using setting 1 (PbO_2_–F + BDD). Meanwhile, 0.67, 14.36, and 2.0 mg L^–1^ were accumulated for the same carboxylic acids, respectively, with setting 2 (BDD + PbO_2_–F) (Figure 6d). Additionally, the formation of salicylic acid (0.13 mg L^–1^), under the same experimental conditions, was attained. Conversely, the electroconversion of MO into carboxylic acids with settings 1 and 2 by applying 45 mA cm^–2^ promoted an increase in their accumulation, achieving 2.22, 28.21, 2.15, 0.34, and 0.16 mg L^–1^ with PbO_2_–F + BDD configuration (Figure 6c), while 1.58, 30.45, 2.32, 0.55, and 0.26 mg L^–1^ with the BDD + PbO_2_–F arrangement (Figure 6f), for formic, malonic, tartaric, acetic, and salicylic acids, respectively.

**Figure 6:**
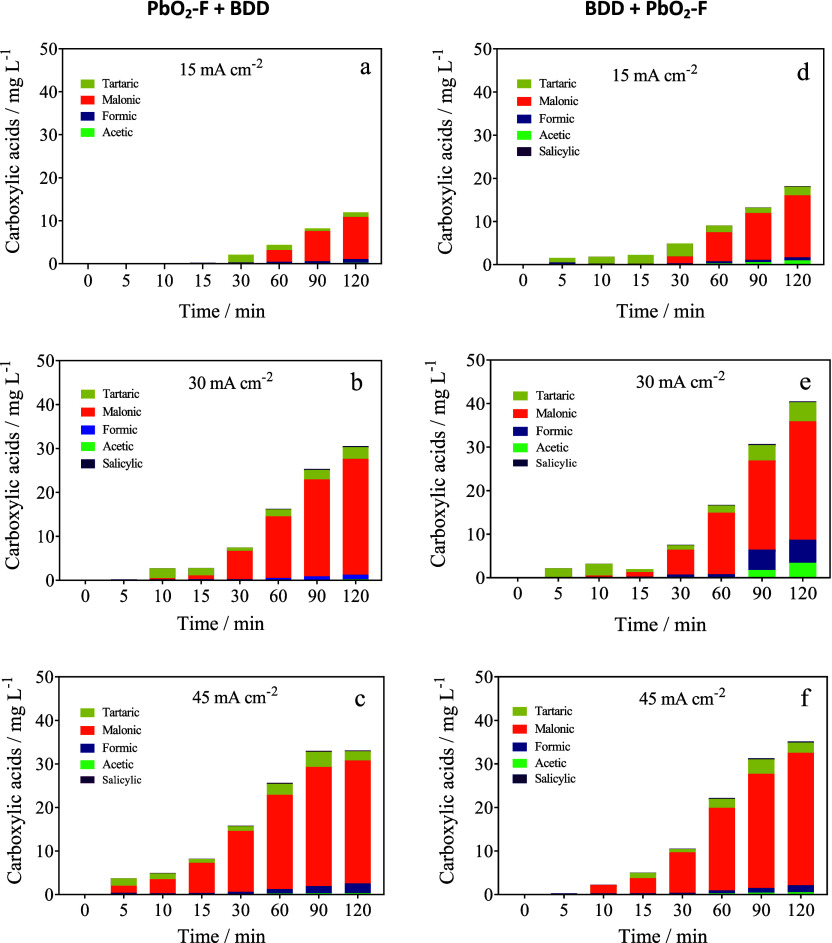
Evolution of the concentration of carboxylic acids as a function of electrolysis time using setting 1 (a,b,c) PbO_2_–F + BDD and setting 2 (d,e,f) BDD + PbO_2_–F. Operating conditions: 0.05 mol L^–1^ Na_2_SO_4_ as the electrolyte; 20 mg L^–1^ MO as the initial concentration, 1 L of synthetic effluent that was recirculated by the divided membrane-type flow reactor at 25 °C to degrade MO and simultaneously produce green H_2_.

As can be seen in Figure 6, malonic acid was preferentially produced under all operating conditions. This organic acid serves as a precursor for polyesters and can be converted into 1,3-propanediol, a compound used in the production of polyesters and polymers. Malonic acid can also be utilized to produce alkyd resins, which are utilized in a variety of coating applications to protect against UV light, oxidation, and corrosion-induced damage. In the coatings industry, malonic acid is utilized as a cross-linker for low-temperature cure powder coatings, which are gaining importance due to their ability to protect heat-sensitive substrates and expedite the coatings process.^86^ In 2014, an estimated amount of about $18.59 billion was allocated to the worldwide automobile coatings market. From 2014 to 2022, this market expanded at a compound annual growth rate of 5.1%.^86^ Therefore, the production of malonic acid from the residual waste is a significant improvement.

Table 1 shows a summary of the yields obtained after 120 min of electrolysis for all the carboxylic acids identified based on %C relative to the initial TOC (1.51 mg L^–1^) of the MO solution used. For this purpose, eq 6 was used, estimating the yields obtained for the identified carboxylic acids:^6,87^

6 $$\eta \left(\%\right)=\frac{\left[\mathrm{Ac}\right]12N_{c}}{{\mathrm{MM}}_{\mathrm{CA}}{\mathrm{TOC}}_{i}}\times 100$$

where, [Ac] is the concentration of the carboxylic acid (mg L^–1^), 12 is the molecular mass of carbon, *N*_c_ is the number of carbon atoms present in the organic acid, MM_CA_ is the molecular mass of the acid (g mol^–1^), and TOC_*i*_ is the total organic carbon content in the initial sample.

In the case of waste valorization, the electrotransformation of a residual effluent into anodic and cathodic valuable compounds represents an advance in electrocatalytic strategies that converge in the circular economy. In fact, a limited number of studies have considered the use of effluents to produce high-value compounds such as carboxylic acids. Some of these investigations are reported in Table 2.

**Table 2:** Selected Examples of Electrochemical Conversion of Biomass into Carboxylic Acids as High Value-Added Products

Wastewater	Experimental	Remarks ([carboxylic acid] = mg L^–1^)	H_2_ production	ref.
Kraft lignin	100 mL of 5 g L^–1^ of kraft lignin with 1 mol L^–1^ NaOH, 2.5 V, 7 h, divided cell (Swiss-Roll cell), nickel foam (5.624 cm^2^) as working and counter electrodes	Acetic acid (210.0), Formic acid (1340.0), Malic acid (8.5)	-	Di Marino et al., (2019)^2^
Glycerol	12.5 mL of effluent of 0.5 mol L^–1^ of glycerol in 2 mol L^–1^ KOH, *j* = 2.5 mA cm^–2^, Ni-boride electrodes using a divided cell, anion exchange membrane	Acetic (24.0) Formic (17031.1), Glyceric (487.9), Glycolic (2129.4), Oxalic (1125.3), Latic (3242.8),	-	Brix et al., (2021)^88^
t-CNSL^a^	250 mL of the t-CNSL solution in 1.0 mol L^–1^ NaOH, open-undivided cell (Nb/BDD as anode and Ti as cathode), *J* = 70 mA cm^–2^, electrolysis time 180 min,	Acetic acid (309.0), Oxalic acid (630.0)	-	Medeiros et al., (2020)^76^
t-CNSL^a^	250 mL of 0.01% of t-CNSL in 1 mol L^–1^ NaOH, 70 mA cm^–2^, 30 min, undivided batch cell, DSA (Ti/TiO_2_RuO_2_IrO_2_), as anode and Ti as cathode	Acetic acid (63.5), Oxalic acid (4.1)	-	Medeiros et al., (2022)^87^
Effluent of washing machine	1000 mL of a real effluent with 0.1 mol L^–1^ Na_2_SO_4_, 60 mA cm^–2^, 150 min, divided membrane-type flow reactor, Nb/BDD as anode and Ni–Fe based SS mesh as cathode	Acetic acid (45.7), Oxalic acid (4.9)	0.8 L	Oliveira et al.,(2023)^38^
Methyl Orange dye	1000 mL of 20 mg L^–1^ of MO with 0.05 mol L^–1^ Na_2_SO_4_, 45 mA cm^–2^, 120 min, divided membrane-type flow reactor, Nb/BDD and PbO_2_ as anodes and Ni–Fe based SS mesh as cathode	Malonic acid (30.46), Tartaric acid (2.32) Formic acid (1.58)	7.0 L (setting 1) and 7.5 L (setting 2)	This work

a Technical cashew-nut shell liquid.

The production of carboxylic acids was successfully accomplished by using nondivided electrochemical reactors as well as active and nonactive anodes.^76^ However, considering the development of an integrated-hybrid process for producing anodic and cathodic valuable compounds from effluents, as well as the enhancement of organic acids’ accumulation, a divided cell was selected.^2,87^ For example, several carboxylic acids (acetic, formic, malic, malonic, oxalic, and succinic acids) were electroproduced when 100 mL of 5 g L^–1^ Kraft lignin in 1 mol L^–1^ NaOH was electrolyzed by applying 142 mA m^–2^ during 420 min in an undivided cell. However, the accumulation of acetic (210.0 mg L^–1^) and formic (1340 mg L^–1^) acids was more selectively attained under alkaline conditions in undivided reactors by using a Ni foam electrode. Another example is the electrolysis of glycerol^88^ with Ni-boride electrodes using a divided cell under alkaline and soft oxidation conditions (12.5 mL of effluent containing 0.5 mol L^–1^ glycerol in 2 mol L^–1^ KOH, *j* = 2.5 mA cm^–2^), significantly improving the accumulation of formic (17031.10 mg L^–1^), glyceric (487.97 mg L^–1^) glycolic (2129.40 mg L^–1^), and oxalic (1125.37 mg L^–1^) acids even when acetic and tartronic acids are also produced. The production of acetic, formic, and oxalic acids was demonstrated when 0.1% biomass effluent was electrolyzed (at 40 mA cm^–2^) with the BDD anode in an alkaline medium (1.0 mol L^–1^ NaOH),^76^ reaching accumulations of about 144, 120, and 75 mg L^–1^, respectively, after 240 min. These concentrations were significantly boosted by increasing the *j*, achieving 630 mg L^–1^ for oxalic acid by applying 70 or 100 mg L^–1^ during 240 min, while the concentration of acetic acid significantly increased to 309 and 281 mg L^–1^, for 70 and 100 mA cm^–2^, respectively, during the first 180 min of electrolysis.^76^ These investigations allowed us to understand that the conversion of waste is feasible, highlighting the importance of the design of the electrochemical reactor and the selection of operating conditions to selectively increase the concentration of organic acids.^89^ Also, the electro-refinery of organics can be extended to other renewable sources or residues, investigating the energy-saving electrochemical green H_2_ production to reduce the energy requirements.^88–93^

### 3.5 Safety Evaluation of PbO_2_-F Electrode

To assess the safety of the PbO_2_–F electrodes, the concentration of Pb elements released from the electrode was determined. After 120 min of electrolysis in a 0.05 mol L^–1^ Na_2_SO_4_ solution (45 mA cm^–2^), the concentration of Pb detected was 0.0057 mg L^–1^, which is well below the effluent discharge limits according to CONAMA (National Environmental Council – Brazil) N^o^ 430/2011, Pb < 0.5 mg L^–1^. Therefore, it can be concluded that the leaching of Pb from the electrode is minimal, and its use is considered safe.

### 3.6 Green Hydrogen Production and Energy Consumption

Due to the use of renewable energy, PV panels described in detail in Section 6 in the SI and Figures S3 and S4, the energy carrier generated is called green H_2_. Figure 7a shows that no effect on the volume of green H_2_ produced was attained, independent of the different electrode arrangements and cell configurations. The volume of H_2_ produced changed linearly as a function of electrolysis time, with a correlation factor *R* ^2^ > 0.9997 in all cases. This means that the experimental values were in good agreement with theoretical production values, which obey Faraday’s law.^94^ Therefore, a greater production of H_2_ is achieved by applying higher *j* values to the system, regardless of the configuration setting used. These results also confirm that the rate of H_2_ production is directly proportional to *j*, being independent of the concentration, nature of the compound, and type of anodic electrode.^74,94–96^ Regarding the cell used in this study, its configuration with two anodes, working in series mode, showed satisfactory results when compared to the results achieved in previous works,^41^ where a single cell (only one anode and one cathode) was used.

**Figure 7:**
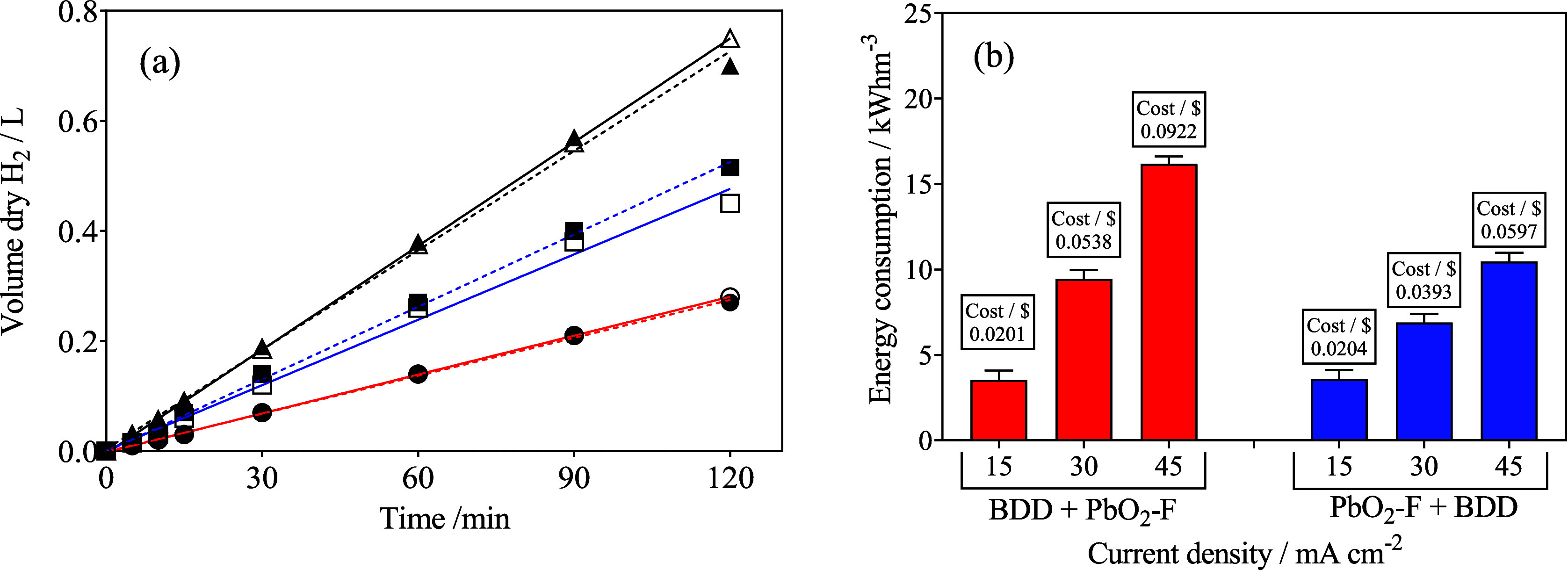
(a) H_2_ production using a Ni–Fe mesh as the cathode in different configurations of the electrochemical cell (solid circles: configuration 1 (PbO_2_–F + BDD), empty circles: configuration 2 (BDD + PbO_2_–F)) by applying (●/○)­15, (●/○) 30, and (▲/Δ) 45 mA cm^–2^; (b) effect of the arrangement of the cells on energy consumption (kWh m^–3^) and cost for the integrated hybrid wastewater||H_2_ cell. Operating conditions: 0.05 mol L^–1^ Na_2_SO_4_ as electrolyte; 20 mg L^–1^ of MO as initial concentration, 1 L of synthetic effluent that was recirculated by the divided membrane-type flow reactor at 25 °C to degrade MO and simultaneously produce green H_2_.

On the other hand, energy consumption (EC) for hydrogen production is an essential parameter to consider in order to reduce economic and environmental impacts. This variable can be determined in terms of kWh m^–3^, according to eq 7

7 $$\mathrm{EC}\;\left({\mathrm{kWh m}}^{-3}\right)=\left(\frac{E_{\mathrm{cell}}\times I\times t}{1000\;V_{s}}\right)$$

where *E*_cell_ corresponds to the average potential difference of the cell (V), *I* is the current intensity (A), *t* is the electrolysis time (h), and *V*_s_ is the solution volume (m^3^). Figure 7b shows the energy consumption values for the different cell configurations by applying different *j*. As observed, an increase in *j* causes an increase in the energy requirements consumed because the cell potential also increases. It is worth mentioning that setting 1 (PbO_2_–F + BDD) showed lower energy consumption at lower *j* (30 mA cm^–2^, approximately 6.38 kWh m^–3^), while for setting 2 (BDD + PbO_2_–F), 9.48 kWh m^–3^ was estimated.

Another important aspect is the economic viability of electrochemical processes, estimating the energy cost for the operation of an integrated hybrid wastewater||H_2_ cell. The calculation of the energy cost (USD m^–3^) was estimated from eq 8, considering that the energy source is a photovoltaic system, based on the costs of the International Renewable Energy Agency (IRENA, 1 kWh = USD 0.0057 (IRENA website).^97^

8 $$\mathrm{Energy cost}\;\left({\mathrm{USD m}}^{-3}\right)=\mathrm{EC}\times 0.557$$

The results in Figure 7b show that the cost for 2 h of system operation is approximately 0.092 and 0.060 USD m^–3^ when 45 mA cm^–2^ is applied, for configurations 1 and 2, respectively. These results clearly demonstrate that the series combination of the divided membrane-type flow reactor, depending on the position of the anodes, is an interesting reactor arrangement to be implemented for the degradation of organic dyes with the simultaneous production of green H_2_, achieving lower energy requirements to attain two integrated processes.

It is well-known that bleaching and finishing account for around 38% of total water use in textile factories, followed by dyeing (16%), printing (8%), boilers (14%), spinning (6%), weaving (9%), and residential and sanitary uses (9%). Water is also used for washing dyed and printed fabrics and for cleaning printing machines and dyeing the MO, resulting in a total vessel. Then, the daily consumption of water in an average-sized textile plant producing about 8000 kg of fabric per day is about 1.6 million liters. The total costs for the treatment of wastewater at the biological wastewater pretreatment plant are about €0.5 per 1 m^3^ in case that the resulting pretreated wastewater has a COD of at least 800 mg L^–1^. Meanwhile, green H_2_ now costs USD $4–6/kg (€3.72–5.58/kg) by using clean water.^97^ Considering these costs, the hybrid-integrated approach could be economically sustainable because the volume of water consumed and treated per day by the textile industry would have a cost of about €800 and €150 for biological and electrochemical treatments (considering the IRENA prices and the energy requirements obtained in this study), respectively, generating 88,335 kg of green H_2_ simultaneously.

## 4 Conclusions

The electro-refinery of organic compounds and energy generation from the electrochemical degradation of MO were successfully achieved in both electrochemical arrangements under the experimental conditions adopted, representing a sustainable alternative for the conversion of textile industrial effluents into value-added chemical products. The efficiency of the process was affected by the anode arrangement since it determines how easily the ^•^OH radicals are supplied to the system and how these oxidizing species react with the organic pollutants, promoting their degradation. Configuration 1 (PbO_2_–F + BDD) by applying 45 mA cm^–2^, after 120 min of reaction, achieved a COD removal efficiency of 42.5% and a H_2_ production volume of 0.70 L, with 10.47 kWh m^–3^ of energy consumed. Meanwhile, configuration 2 (BDD + PbO_2_–F), under the same conditions, achieved 47.5% COD removal, 0.75 L of H_2_ and an energy consumption of 16.18 kWh m^–3^. A variety of carboxylic acids were detected, although malonic acid is the one with the highest concentration. From an economic point of view, the daily water consumption in a textile factory is substantial, so integrated hybrid electrochemical treatment (wastewater||H_2_) may be the best way to overcome the barriers related to the high costs normally attributed to industrial effluent treatments.

## Supplementary Material



## Notes

The characterization performed for the PbO_2_–F electrode, as well as the analytical techniques used to monitor the dye degradation parameters; the mass transport characterization of the electrochemical reactor was reported, which is fundamental for determining the favorable combinations of arrangements; kinetic analysis of degradation and the photovoltaic arrangement description as well as its performance to drive electrochemical reactor (PDF)
