# Supplementary material for: Sustainable Photovoltaic-Powered Series Electrochemical Reactors Using Nonactive Electrocatalytic Materials for Electro-Refinery in Organics and Energy Production
Source: ACS Omega. 2025 Jul 14;10(29):31466–79. doi: 10.1021/acsomega.5c01329 (PMC12311666; doi:10.1021/acsomega.5c01329)
Supplement: Supplementary file 1 [file ao5c01329_si_001.pdf]

Supporting Information

Sustainable photovoltaic-powered series electrochemical reactors using non-active electrocatalytic materials for electro-refinery in organics and energy production

Rainy Alves de Sousa[1,2], Livia N. Cavalcanti[1], Amanda D. Gondim[1], Jussara Câmara Cardozo[1,*], Marco A. Quiroz Alfaro[1], Carlos A. Martínez-Huitle[1,3,*], Elisama Vieira dos Santos[1,3,*]

[1] Renewable Energies and Environmental Sustainability Research Group, Institute of Chemistry, Federal University of Rio Grande do Norte, Campus Universitário, Av. Salgado Filho 3000, Lagoa Nova, CEP 59078-970, Natal, Rio Grande do Norte, Brazil.

[2] Human Resources Program of the National Agency for Petroleum, Natural Gas and Biofuels – PRH-26-ANP, Graduate Program in Chemical Engineering - PPGEQ, Lagoa Nova, Natal/RN, 59078-970, Brazil

[3] National Institute for Alternative Technologies of Detection, Toxicological Evaluation and Removal of Micropollutants and Radioactives (INCT–DATREM), Institute of Chemistry, UNESP, P.O. Box 355, 14800 900 Araraquara, SP, Brazil

Corresponding author e-mails: camarajussara@gmail.com, carlosmh@quimica.ufrn.br, elisama.vieira@ufrn.br

1. Characterization of F-doped PbO_2 films on Ti substrate

The crystalline structure of the PbO_2-F films was analyzed by X-ray diffraction (XRD) on a Shimadzu XRD-6000 diffractometer with Cu Kα source (λ = 1.5418 Å) operated at 30 kV and 30 mA. The morphology of the PbO_2-F electrode were investigated by scanning electron microscopy (SEM; Carl Zeiss, Auriga, Oberkochen, Germany), and an energy dispersive spectroscopy analysis which was executed by a scanning electron microscope (EDS, Bruker, XFlash detector, Billerica, MA, USA). Electrochemical characterizations including linear sweep voltammetry (LSV) were measured with an Autolab PGSTAT302N (Metrohm) controlled with NOVA software using a three-electrode cell, with Ag/AgCl (3.0 mol L^{-1} KCl) as reference electrode, a Pt wire as an auxiliary electrode, while PbO_2-F or BDD materials were used as the working electrode. LSV measurements were used to analyze and compare the potentials at which the oxygen evolution reaction (OER) is attained on the working electrodes. The measurements were performed in a 0.05 mol L^{-1} Na_2SO_4 solution at 25 °C using a sweep rate of 50 mV s^{-1}.

2. Analytic methods

The concentration of the MO was followed up by differential pulse voltammetry (DPV) in an Autolab PGSTAT302N (Metrohm) controlled with NOVA software, using a three-electrodes cell, with Ag/AgCl (3.0 M KCl), a Pt wire and BDD with a geometric area of 1 cm^2, as the reference, auxiliary and working electrodes, respectively [1,2]. Some parameters were established for the procedure, such as equilibrium time = 5 s; modulation amplitude = 0.05; time interval = 0.5 s; initial potential = 0 V; final potential = +1.8 V; step potential = 25 mV. A calibration curve was obtained by analyzing the peak intensity of the DPV profiles of MO standard solutions as a function of the analyte concentration, which ranged from 3 to 25 mg L^{-1} in 0.05 mol L^{-1} Na_2SO_4. COD measurements were

determined using the protocol described elsewhere [3,4]. The evolution of acetic, formic, malonic, tartaric and salicylic acids as final organic by-products of MO dye degradation was monitored using a DIONEX ICS-2000 ion chromatograph, equipped with a Dionex AS set. The separation was carried out on a 2 mm Dionex IonPac AS19 column (250 mm, 2 mm ID) used with a Dionex AG19 guard (50 mm, 2 mm ID), coupled to a 2 mm Dionex ASRS300 suppressor. Hydroxide eluent gradients were generated in-line using the Dionex RFIC-EG KOH cartridge at a flow rate of 0.25 mL min^{-1}. Standard solutions of inorganic anions were purchased as anion concentration standards of different ppm from Aldrich and were diluted as necessary with milli-Q water. Water treated with a Millipore (Bedford, MA, USA) Milli-Q system was used to prepare the standard solutions and eluents. Decolorization was monitored by measuring the absorbance of the peak at a wavelength of 464 nm using a Shimadzu UV 1800 spectrophotometer during the tests and then DFZ-visual color number (German abbreviation of DurchsichtFarbZahl) was done according to Equation S1 following the DIN EN 7884:2012 method [5,6].

$$DFZ_y = 100\,\frac{E_y}{d} \tag{S1}$$

where *Ey* is the absorbance at a *y* wavelength and d is the cell path length in cm.

To determine persulfate production, 1 mL standard solution of persulfate, 0.2 g $NaHCO_3$ (to avoid air-oxidation of iodide) and 4 g KI were added to 40 mL pure water in a 40 mL colorimetric tube. Thereafter, the solutions were mixed and allowed to equilibrate for 15 min. The analytical wavelength was fixed at 352 nm [7]. The concentrations of persulfate were compared with electroanalytical determinations by using Au sensor as described elsewhere [8]. It is important to remark that no pre-treatment of samples is required.

The Pb determinations were carried out by inductively coupled plasma atomic emission spectrometry using an ICP-AES (Thermo Fisher Scientific, Bremen, Germany), model iCAP 6300 Duo, with axial and radial views, and simultaneous CID (Charge

Injection Device) detector. Commercial argon (White Martins-Praxair) with a purity of 99.996 % was used to purge the optics, produce the plasma and was used as a nebulizer and auxiliary gas. The sample introduction system used a Burgener Miramis nebulizer and a cyclonic nebulizer chamber. In this system, the sample was pumped into plasma using a peristaltic pump attached to the equipment and its flow rate was controlled by the program (iTeva - Thermo Scientific). RF source power, 1150 W; nebulizer gas flow, 0.75 L min^{-1}; auxiliary gas flow, 0.5 L min^{-1}; stabilization time, 15 seconds; applied wavelength: 220.353 nm.

3. Electroanalytical measurements to quantify MO

Using DPV with BDD material, an electrochemical sensing approach for quantifying MO was developed during its degradation treatment using a PEM-type flow electrochemical reactor, in series mode, powered by photovoltaic panels. DPV technique has been demonstrated to be reliable due to its sensitivity and simplicity [2], therefore, it is a power tool to combine with other electrochemical technologies. To aim this, a calibration curve was constructed at different concentrations of MO (0.5-25 mg L^{-1}) in 0.05 mol L^{-1} Na_2SO_4, as shown in Fig. S1. Analyzing the voltammetric curves in the potential range between +0.4 V and +1.0 V, a well-defined voltammetric signal was registered at +0.75 V, where, as can be seen in Fig. 1S, the peak current has an excellent linear relationship with the concentration of MO. Analyzing the DPV curves in Fig. S1, it was possible to obtain a linear equation with a regression coefficient of approximately 0.995 (inset Fig. S1). The sensitivity is given by the slope of the calibration line, 1.08×10^{-6} mg L^{-1}; while the precision can be expressed as the relative standard deviation, RSD, with an acceptable value of 3.1% (n = 8), and the limits of detection (LOD) and

quantification (LOQ) of about 1.02 mg L^{-1} and 3.09 mg L^{-1}, respectively, were also obtained.

Furthermore, the residuals of the regression were distributed in a random manner around zero (as shown in the second inset of Fig. S1), which enabled a visual examination to confirm the lack of substantial nonlinearity. According to IUPAC recommendations and prior research in the field [9,10], these methods (LOD and residuals) can control both false positive and false negative errors ($\alpha = \beta = 0.05$) [11]. It is crucial to note that supplementary calibration curves were acquired on separate days to validate the BDD electrochemical sensor's excellent stability, as no changes in the statistical values (approximately 1.92% relative standard deviation (RSD)) were detected. Subsequently, the outcomes unequivocally demonstrated that MO can be quantified utilizing DPV in conjunction with a BDD sensor, thereby enabling the monitoring of the compound's degradation progression throughout the electrolysis process.

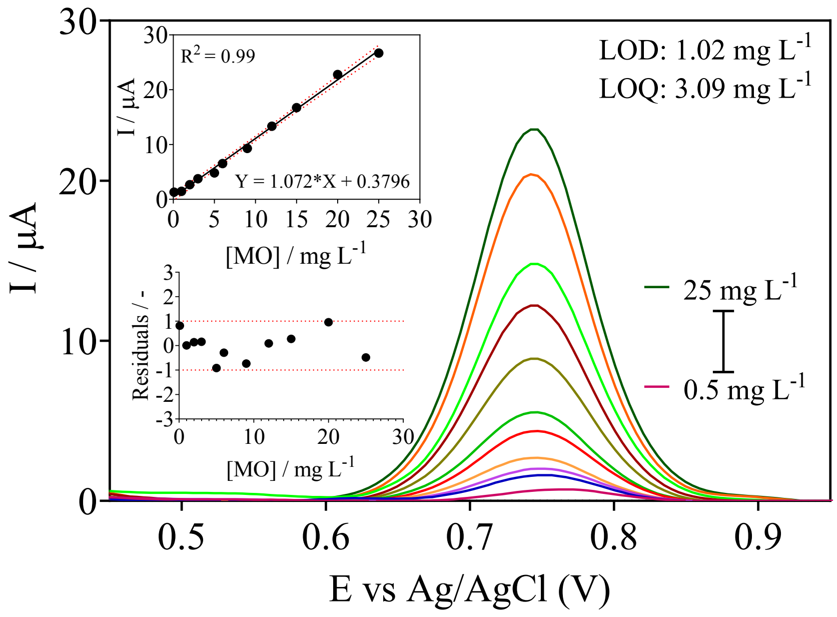

Figure S1. DPV curves for electrochemical sensing approach in 0.05 mol L^{-1} Na_2SO_4 by using the successive standard MO additions method. Working electrode: BDD (1 cm^2),

Counter electrode: Pt wire, Reference electrode: Ag/AgCl 3M; Scanning rate: 50 mVs^{-1}. Inset 1: Analytical curve based on the linear relationship between current peak and concentration. Inset 2: residuals of the regression (analytical curve) as a function of MO concentration.

4. Electrolyzer mass-transport characterization

The electrochemical reactor is a critical factor influencing the performance of EO of organic pollutants. For effective removal of pollutants, different electrochemical reactors have been employed (flow cells with parallel electrodes, flow plants with a three-phase three-dimensional electrode, bipolar trickle tower reactor)[12]; In fact, the assembling of electrochemical flow cells in series to be used for removing dyes has been already investigated by our group[13,14] to determine the favorable arrangement combinations (nature of electrode (active or non-active, cell-electrode positions, current densities, organic removal load, energy requirements and so on). Therefore, the experimental conditions tested here were chosen based on our previous investigations.

Chemicals were of the highest quality commercially available and used without further purification. Aqueous solution of potassium ferricyanide ($K_4Fe(CN_6)$) with a concentration in the range of 20-80 mmol L^{-1} in 0.5 mol L^{-1} NaOH was electrolyzed in each one of the flow reactors and the limiting current for the cathodic reduction of $K_4Fe(CN_6)$ was measured [15]. To determine the limiting currents, polarization curves were plotted by increasing the voltage stepwise and registering the corresponding current [16] (Fig. S2). Subsequently, the relationship between limiting currents and $K_4Fe(CN_6)$ concentrations was obtained (inset in Fig. S2) and the mass transport coefficient was determined by using Eq. S2.

$$k_m = \frac{i_L}{zFAC_\infty} \qquad \text{(S2)}$$

where k_m is the mass-transfer coefficient (m s^{-1}), i_L is the electrolysis limiting current (A), z is the electrons transfer in the redox reaction (for this redox pair, 1), F is the Faraday constant (96487 C mol^{-1}), A is the electrode surface area (m^2) and C_∞ is the bulk species concentration (mol dm^{-3}).

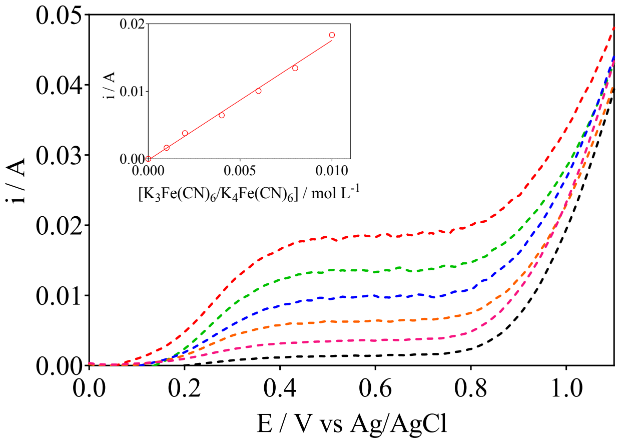

Figure S2. Polarization curves for the km characterization of the electrochemical cell using ferro/ferricyanide protocol (i.e., 1-10 mmol L^{-1}) in 0.5 mol L^{-1} NaOH. Inset: Variations of limiting current as a function of ferro/ferricyanide concentration.

The value of the k_m was subsequently used together with the initial value of COD of the MO effluent, as required by Eq. S3, to estimate the limiting current (I_{lim}), as follows:

$$I_{lim(t)} = 4FAk_mCOD_{(t)}$$

(S3)

where $I_{lim(t)}$ is the limiting current (A) at a given time t, 4 the number of exchanged electrons, F the Faraday's constant, A the electrode area (m^2), k_m the average mass transport coefficient in the electrochemical reactor (m s^{-1}) and $COD_{(t)}$ the chemical oxygen demand (mol O_2 m^{-3}) at a given time t.

5. Kinetic analysis

To study the kinetics of the overall reaction involved in the disappearance of MO and intermediates by direct EO, was considered the decay of COD under different *j*. Results given in Figs. 4a and 4b, in the main text, were further analyzed using kinetic equations related to different reaction orders. Good linear plots were only obtained at all *j*, when the COD concentration decays were fitted to a pseudo-first-order reaction ($In(COD_0/COD_t)$ vs time). As can be seen in the insets of Figs. 4a and 4b in the main text, this behavior was found for all *j* for both configuration arrangements of PEM-type flow electrochemical reactor, in series mode (setting 1 (PbO_2-F + BDD) and setting 2 (BDD + PbO_2-F)) driven by a solar PV-battery system, up to 120 min, giving the pseudo-rate constant (*k*) values reported in the main text. This suggests that the reaction rate increases when an increase on the *j* is attained, and that, the concentration of all oxidizing species ($^{\bullet}OH$ and sulphate-based species) depends on *j* used. However, these figures suggest that the $^{\bullet}OH$ are also consumed to favor oxygen evolution.

6. PV panels to supply electrical energy to the electrochemical reactor

Two Canadian CS6U-325p polycrystalline silicon solar PV modules connected in series with a combined peak output of 640Wp were used to control electrochemical experiments (Fig S3). The solar panels were installed on the roof of the Núcleo de Estudos em Petróleo e Energias Renováveis (NUPER) at the Federal University of Rio Grande do Norte Natal in Brazil (W 35° 12′, S 05° 54′), inclined at 5°, and facing south (20 °W). For optimal electric energy extraction from solar cells, an MPPT (VictronBluesolar 150/45-MC-4) was connected between the two 12 V batteries (Solar Freedom, 12 V/240 Ah each) and the PV modules. To control the current supply to the electrochemical reactor, the batteries

were directly connected to the MIMIPA MPL-3305 M triple power DC generator, which was used to apply j. Fig. S4 shows the amount of solar radiation that was observed during the experimental period (on a typical sunny day – Fig. S4a) as well as the amount of energy (current intensity) that was produced by the PV cells (average intensity of solar irradiation: about 20,000 kJ m^{-2} per day, for 100 days (Fig. S4b)). These sunny weather conditions are achieved practically every day in the city of Natal, due to the proximity to the equator. Meanwhile, Fig. 4Sc shows the instantaneous current profile produced by the solar PV modules, with the electricity used to charge the batteries immediately. The intensity of the current increases steadily during the morning in accordance with the intensity of solar irradiation, reaching a daily peak of 16.8 A around midday and lasting about 3.5–4 hours a day. Furthermore, the time interval (from 8.5 to 10.0 h) of no current during the night is clearly visible (Fig. 3Sc). When the irradiation intensity is greater than 15,000 kJ m^{-2}, the solar PV modules can produce current intensities of more than 10 A for 8–9 h per day with an almost constant voltage of 27 V. This suggests that even in the early morning there is sufficient solar irradiation to allow for the best performance/output from the PV modules and subsequent battery recharging. The electrical energy stored in the batteries is used to supply the necessary current to the electrochemical system during the decontamination of the effluent while the simultaneous electrochemical production of H_2 is achieved. Due to the use of renewable energy, the energy carrier generated is called green H_2.

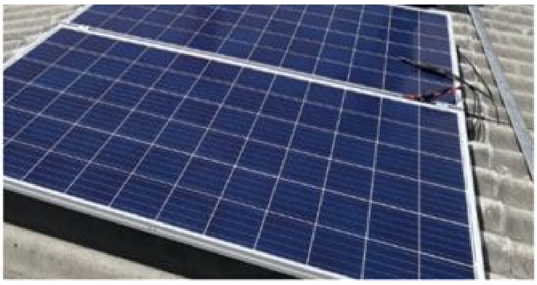

Figure S3. Installation of polycrystalline silicon modules (model Canadian CS6U-325p) interconnected in serial array, providing 640 Wp of power, which were installed at the Petroleum and Renewable Energy Studies Center at the Federal University of Rio Grande do Norte (W 35° 12′, S 05° 54′), with an inclination of 5° to the south and west orientation (20 °W).

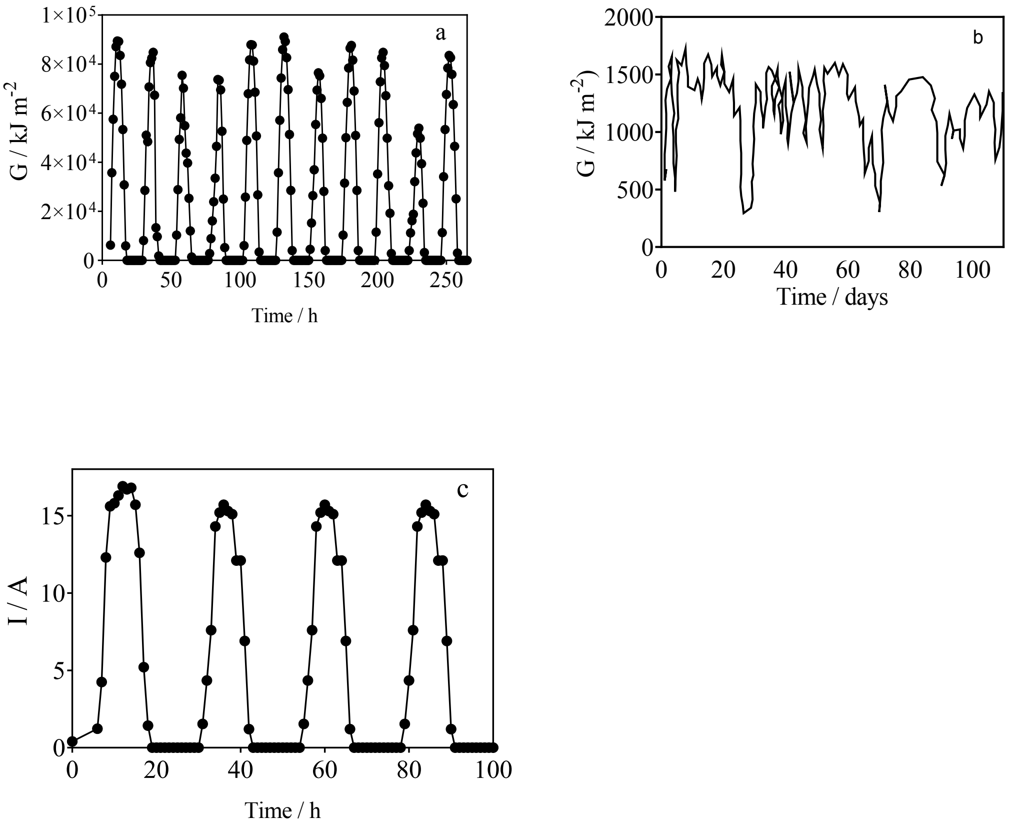

Figure S4. Example of the evolution of (a) solar irradiation intensity, (b) average solar irradiation intensity per day and (c) total current intensity generated from the PV cells.

References

(1) Karla C. F. Araújo; de Oliveira Silva, Karyn Nathallye, Mayra K. S. Monteiro, djalma ribeiro da Silva, Marco A. Quiroz, Elisama Vieira dos Santos, C. A. M.-H. Towards Use of Persulfate Electrogenerated at Boron Doped Diamond Electrodes as Ex-Situ Oxidation Approach: Storage and Service-Life Solution Parameters. *J Electrochem Soc* **2022**, *169*, 033506. https://doi.org/10.1149/1945-7111/ac59f8.

(2) Karyn N. O. Silva, Karla C. F. Araújo, Djalma R. da Silva, Carlos A. Martínez-Huitle, E. V. dos S. Persulfate-Soil Washing: The Green Use of Persulfate Electrochemically Generated with Diamond Electrodes for Depolluting Soils. *Journal Journal of Electroanalytical Chemistry* **2021**.

(3) Cardozo, J. C.; Barbosa Segundo, I. D.; Galvão, E. R. V. P.; da Silva, D. R.; dos Santos, E. V.; Martínez-Huitle, C. A. Decentralized Environmental Applications of a Smartphone-Based Method for Chemical Oxygen Demand and Color Analysis. *Scientific Reports 2023 13:1* **2023**, *13* (1), 1–13. https://doi.org/10.1038/s41598-023-37126-9.

(4) de Castro, C. M.; Olivi, P.; de Freitas Araújo, K. C.; Barbosa Segundo, I. D.; dos Santos, E. V.; Martínez-Huitle, C. A. Environmental Application of a Cost-Effective Smartphone-Based Method for COD Analysis: Applicability in the Electrochemical Treatment of Real Wastewater. *Science of The Total Environment* **2023**, *855*, 158816. https://doi.org/10.1016/J.SCITOTENV.2022.158816.

(5) Silva, J. C. O. da; Solano, A. M. S.; Segundo, I. D. B.; Santos, E. V. dos; Martínez-Huitle, C. A.; Silva, D. R. da. Achieving Sustainable Development Goal 6 Electrochemical-Based Solution for Treating Groundwater Polluted by Fuel Station. *Water 2022, Vol. 14, Page 2911* **2022**, *14* (18), 2911. https://doi.org/10.3390/W14182911.

(6) Silva, L. G. M.; Moreira, F. C.; Souza, A. A. U.; Souza, S. M. A. G. U.; Boaventura, R. A. R.; Vilar, V. J. P. Chemical and Electrochemical Advanced Oxidation Processes as a Polishing Step for Textile Wastewater Treatment: A Study Regarding the Discharge into the Environment and the Reuse in the Textile Industry. *J Clean Prod* **2018**, *198*, 430–442. https://doi.org/10.1016/j.jclepro.2018.07.001.

(7) Liang, C.; Huang, C. F.; Mohanty, N.; Kurakalva, R. M. A Rapid Spectrophotometric Determination of Persulfate Anion in ISCO. *Chemosphere* **2008**, *73* (9), 1540–1543. https://doi.org/10.1016/j.chemosphere.2008.08.043.

(8) José, J. E.; D. Gondim, A.; Vieira dos Santos, E.; Martínez-Huitle, C. A. Innovative and Efficient Electroanalytical Approach for Determining Persulfate in Aqueous Solutions Using a Gold Electrode. *Chemosphere* **2023**, *344*, 140263. https://doi.org/10.1016/J.CHEMOSPHERE.2023.140263.

(9) Desimoni, E.; Brunetti, B. About Estimating the Limit of Detection of Heteroscedastic Analytical Systems. *Anal Chim Acta* **2009**, *655* (1–2), 30–37. https://doi.org/10.1016/j.aca.2009.09.036.

(10) Miller, J. N. Basic Statistical Methods for Analytical Chemistry. Part 2. Calibration and Regression Methods. A Review. *Analyst* **1991**, *116* (1), 3–14. https://doi.org/10.1039/AN9911600003.

(11) Danzer, K.; Currie, L. A. Guideline for Calibration in Analytical Chemistry— Part 1. Fundamentals and Single Component Calibration. *Pure and Applied Chemistry* **1998**, *70* (4), 993–1014. https://doi.org/10.1351/pac199870040993.

(12) Brillas, E.; Martínez-Huitle, C. A. Decontamination of Wastewaters Containing Synthetic Organic Dyes by Electrochemical Methods. An Updated Review. *Appl Catal B* **2015**, *166–167*, 603–643. https://doi.org/10.1016/j.apcatb.2014.11.016.

(13) Fajardo, A. S.; Martins, R. C.; Silva, D. R.; Quinta-Ferreira, R. M.; Martínez-Huitle, C. A. Electrochemical Abatement of Amaranth Dye Solutions Using Individual or an Assembling of Flow Cells with Ti/Pt and Ti/Pt-SnSb Anodes. *Sep Purif Technol* **2017**, *179*, 194–203. https://doi.org/10.1016/j.seppur.2017.01.029.

(14) Fajardo, A. S.; Martins, R. C.; Martínez-Huitle, C. A.; Quinta-Ferreira, R. M. Treatment of Amaranth Dye in Aqueous Solution by Using One Cell or Two Cells in Series with Active and Non-Active Anodes. *Electrochim Acta* **2016**, *210*, 96–104. https://doi.org/10.1016/j.electacta.2016.05.102.

(15) Quiroz, M. A.; Martínez-Huitle, U. A.; Martínez-Huitle, C. A. Mass Transfer Measurements in a Parallel Disk Cell Using the Limiting Current Technique. *J Mex Chem Soc* **2005**, *49* (3), 279–283.

(16) Brito, C. N.; Ferreira, M. B.; de Moura Santos, E. C. M.; Léon, J. J. L.; Ganiyu, S. O.; Martínez-Huitle, C. A. Electrochemical Degradation of Azo-Dye Acid Violet 7 Using BDD Anode: Effect of Flow Reactor Configuration on Cell Hydrodynamics and Dye Removal Efficiency. *J Appl Electrochem* **2018**, *48* (12), 1321–1330. https://doi.org/10.1007/s10800-018-1257-4.
